# Care of the Mouth in Sick Persons

**Published:** 1894-10

**Authors:** 


ARTICLE III.

### CARE OF THE MOUTH IN SICK PERSONS.

Rosenbach (*Zeit fur Krankenpflege*, April, 1894) says that in many illnesses there is almost sure to be secondary trouble in the mouth, if preventive measures be not taken. A warning sign is dryness and redness of the tongue and mucous membrane of the mouth, with difficulty in swallowing; further signs are an evil odor from the mouth, coated tongue and gums, bleeding of the gums, etc. Just as special care of the mouth is required in patients with carious teeth, smokers and chewers of tobacco, so it is also in the case of unconsciousness or paralyzed persons; patients with fever or suffering from chronic digestive complaints; those taking medicines, such as mercury or iodides, or who on account of general weakness, have to take strong alcoholic drink; but perhaps the most important class of those in whom special care of the mouth must be taken are patients with fever. Parasites are always present in the mouth, but it is only when the tissues are weakened that they undergo invasion by these parasites. There is nothing which one can do for sick persons which is unimportant, and by neglect in the care of the mouth convalesence may be retarded.

Rosenbach concludes with the following rules:

1. Patients with good digestive powers, free from fever, and with no loss of consciousness require no more than the ordinary care of the mouth.

2. In children and very old patients the less solid food taken the greater should be the care with the mouth. They should rinse the mouth out several times a day with lukewarm water containing a little common salt, tincture of myrrh, or eau de cologne added to stimulate secretion. When there is a tendency to bleeding of the gums, or when the teeth are bad, a pinch of powdered boric acid may be twice daily rubbed in between the lips and gums. Patients with false teeth should remove their false teeth when, owing to loss of appetite or chronic gastric disturbance, they cannot take solid food.

3. In patients with partial loss of consciousness the mouth should be examined several times a day for small sores, such as may arise from the pressure of the teeth on the lips, etc. Such sores should be powdered with a little boric acid or chlorate of potassium, and the cracks at the corners of the lips heal quickly if dried with a clean towel and treated with boric acid or vaseline. The mucous membrane may be stimulated by wiping the tongue and mouth and pressing on the tongue with a moist towel every two or three hours; if necessary, the hinder part of the tongue should be cleansed with a wad of cotton-wool fastened to a stem. If the patient sleep with the mouth open, the air in the room must be kept moist; a moistened layer of muslin laid on the mouth may be of some service.

4. Patients with fever should have something to drink—cold water or weak lemonade—at least every hour; one must not wait until the patient asks for drink. Besides preventing dryness, the fluid maintains the activity of the glands and the whole function of the mucous membrane. Many patients are prevented from drinking by a painful, dry, and cracked condition of the lips, and therefore all feverish patients should from the commencement of their illness have their lips rubbed several times a day with vaseline or fat. In protracted cases of fever the mouth may also be swabbed out with oil, fat, or greatly-diluted glycerin.—*British Medical Journal.*

